# Auxiliary Graph for Attribute Graph Clustering

**DOI:** 10.3390/e24101409

**Published:** 2022-10-02

**Authors:** Wang Li, Siwei Wang, Xifeng Guo, Zhenyu Zhou, En Zhu

**Affiliations:** 1 School of Computer, National University of Defense Technology, Changsha 410000, China; 2 School of Cyberspace Science, Dongguan University of Technology, Dongguan 523808, China

**Keywords:** clustering, auxiliary graph, graph networks, attribute graph

## Abstract

Attribute graph clustering algorithms that include topological structural information into node characteristics for building robust representations have proven to have promising efficacy in a variety of applications. However, the presented topological structure emphasizes local links between linked nodes but fails to convey relationships between nodes that are not directly linked, limiting the potential for future clustering performance improvement. To solve this issue, we offer the Auxiliary Graph for Attribute Graph Clustering technique (AGAGC). Specifically, we construct an additional graph as a supervisor based on the node attribute. The additional graph can serve as an auxiliary supervisor that aids the present one. To generate a trustworthy auxiliary graph, we offer a noise-filtering approach. Under the supervision of both the pre-defined graph and an auxiliary graph, a more effective clustering model is trained. Additionally, the embeddings of multiple layers are merged to improve the discriminative power of representations. We offer a clustering module for a self-supervisor to make the learned representation more clustering-aware. Finally, our model is trained using a triplet loss. Experiments are done on four available benchmark datasets, and the findings demonstrate that the proposed model outperforms or is comparable to state-of-the-art graph clustering models.

## 1. Introduction

The attribute graph data is ubiquitous in the real-world. For example, data from social networks [1], citation networks [2], protein-protein interaction networks [3]. For the lack of labeled data, there exists a need to divide data into groups.

In the early days, graph clustering methods used only structure information for network embedding. Utilizing structure information, some methods [4,5] based on random walk implement representation learning by maximizing the probability of cooccurrence of node pairs. Recently, refs. [6,7,8] suggest mining meaningful features from networks with BDM (Block decomposition method). For example, by BDM, ref. [6] obtain graph motif complexity for network clustering. Removing a minimum subset of edges, ref. [7,8] can obtain the desired clusters with minimum loss of information contribution, which is calculated by algorithmic complexity obtained from BDM. Along with the development of deep models, plenty of deep clustering models have emerged [9,10,11,12,13,14]. However, the conventional deep clustering models focus on investigating Euclidean structure data. For example, data of faces, data of animals, data of vehicles. Unlike Euclidean structure data, the relationships between nodes in the graph have nothing to do with their positions in space. For this reason, the traditional deep models cannot handle both the attribute and structure of graph data properly. Recently, the question of how to exploit both graph structure and node attribute sufficiently has attracted more and more attention in clustering tasks. Graph Convolutional Network (GCN) [2] is a powerful model to meet the need mentioned above. A great number of graph clustering models based on GCN have been developed. Inspired by AutoEncoder, Graph auto-encoder (GAE) [15] implements representation learning in an encoder-decoder mechanism. Following GAE, ARGE [16] improves representation learning by introducing an adversarial training module. MGAE [17] proposes to exploit the interplay between node attribute and structure information. GAT [18] introduces an attention mechanism to specify different weights to different neighbors. Following GAT, ref. [19] aggregates its neighbors by learning an attention mechanism in an unsupervised way. SDCN [20] is a deep model that can alleviate the impact of over-smoothness by fusing embeddings from different modalities. Based on SDCN, DFCN [21] improves performance by integrating global structure information into local structure information.

To some degree, these GCN based models exploit structure information in different ways and achieved noticeable improvements. However, we found that there are three kinds of cases that lead to a sub-optimal performance: (1) Methods that ignore global structure completely. (2) Methods that have taken global structure into consideration but trained with only the guidance of given graph structure. (3) Methods that ignore the guidance of the structure. All these mentioned methods fail to exploit the global structure appropriately and lead to a sub-optimal performance consequently.

To solve this issue, unlike those shallow models mentioned before [4,5,6,7,8], we propose a deep graph clustering model termed Auxiliary Graph for Attribute Graph Clustering. In particular, we construct an additional graph as a supervisor based on the similarity between nodes in their raw feature space. However, the newly constructed graph is rife with erroneous relationships due to the underlying noise in the raw data. To mitigate the impact, we employ a filtering technique to choose a certain number of nodes closest to the target nodes. We retain the relationships between each target node and a predefined number of neighbors who can be considered somewhat dependable. Assuming that the remaining relationships are untrustworthy, they are disregarded. We combine embeddings from various layers to generate representations that are highly discriminative. Finally, we have created a training technique that incorporates both reconstruction loss and clustering loss. On the one hand, we optimize our model by forcing it to reconstruct a graph that can approximate both the pre-defined graph and the auxiliary graph. On the other hand, we employ a clustering-oriented optimization whose efficacy has been thoroughly proved. In the former scenario, these two types of rebuilding are complementary. In the latter case, the clustering-friendly model enables learned representations to facilitate the clustering operation.

Our contributions are summarized as follows:

- We build an auxiliary graph to reveal the relationships that were missed by the given graph. With the supervising of both auxiliary graph and given graph, the learned representations are improved to be more reliable.
- The optimization by clustering loss based on fusing embeddings from multiple layers facilitates both the discriminativeness and the clustering-awareness of representations.
- Extensive experiments on four popular benchmark datasets are conducted and the results validate the superiority of our method over the state-of-the-art methods.

## 2. Related Works

Deep clustering has always attracted extensive attention. During the past few years, plenty of deep clustering models have emerged [9,11,13,22,23,24,25,26,27,28,29,30]. Among them, AutoEncoder is a basic DNN model that is widely used for subsequent deep clustering models. In DEC [11], a target distribution is designed to prevent large clusters from distorting hidden feature space, which alleviates the impact of data imbalance. Inspired by [29], IDEC [9] improved DEC by introducing the optimization of reconstruction. Training by reconstructing the input data can keep the local structure-property for embeddings. DSC [22] also introduces an auto-encoder framework to the subspace clustering module. The auto-encoder module can learn a non-linear mapping that facilitates subspace clustering. DMC [30] keeps the local structure by minimizing the distance between the target point and its K-nearest neighbors. At the same time, it also constructs a clustering-friendly objective that improves representations. By forcing the embeddings from the noisy encoder to approximate that from a clean encoder, DEPICT [24] improves the robustness of representations. Although effective, deep clustering models neglect the information from graph structure, which contains a wealth of information that can improve representation learning greatly.

Recently, GCN-based deep clustering models have gained much attention. And an abundance of excellent models have been proposed [15,16,19,20,21,31,32,33,34]. Ref. [15] designed an encoder-decoder framework that based on graph convolution network (GAE) and its variation (VGAE) that was based on VAE [31]. As an unsupervised graph-based representation learning method, it is popular for the tasks of clustering. AGC argues that each graph has its distinct structure, and it is unreasonable to perform clustering tasks on different graphs by aggregating neighbors with a fixed neighborhood. Instead of keeping a fixed neighborhood for each graph, AGC proposed measurement for choosing a proper scale of the neighborhood. In DAEGC [19], neighbors are not equally important to the target nodes. It can capture the importance of neighbors for the target node by an attention network. Some develop different training schemes to improve clustering performance. MGAE [17] corrupts node features by a pre-defined probability to disturb the information so that the interaction between node content and structures can be reinforced and the representation capacity of the network can be improved. ARGE [16] incorporates an adversarial training scheme into GAE, which can learn a robust representation. Instead of reconstructing the graph only, ref. [35] improved the performance of ARGE by reconstructing both the graph and features. EGAE-JOCAS [32] utilizes K-means and spectral clustering jointly to guide the representation learning and improve performance. Some models [20,21,34] combine deep features of multi-modality to alleviate over-smoothness. In SDCN [20], a GCN module and an auto-encoder module are integrated. Incorporated with representations from the auto-encoder module, GCN is capable of capturing the relationship between nodes of longer distances. DFCN [21] improved SDCN by dynamically integrating features of multi-modality and optimizing with triplet guidance which could generate robust representations. AGCN [34] argues that when fusing features, features from different modalities should not be considered to be of equal importance. It proposed to adaptively fuse features of different modalities at each layer, and again adaptively fuse features of different layers.

Most of the methods mentioned above achieved promising performance in clustering, but few consider that there are plenty of relations that are missed by the given graph structure.

## 3. Proposed Method

**Figure 1:**
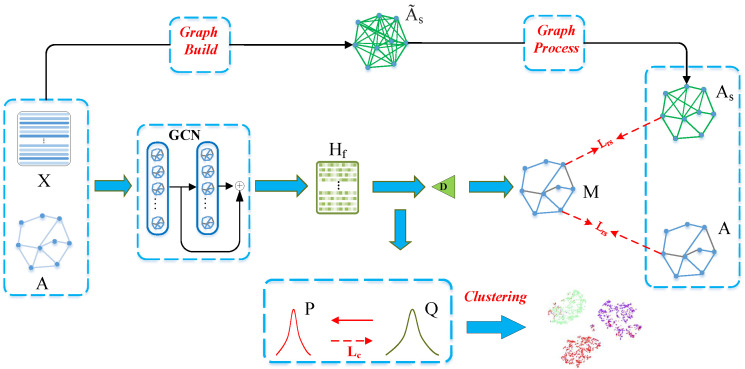
This is the framework of AGAGC. From top to bottom, our model consists of three components: auxiliary graph creation, graph auto-encoder, and clustering procedure. The top section represents the construction of the auxiliary graph $A_{s}$ and consists of two steps: build and process. The construction of an auxiliary graph is a prerequisite to training. As the backbone of the middle section, we employ a graph auto-encoder (GAE). As an encoder, we introduce a GCN module. The encoder accepts as input the feature matrix *X* and the provided graph *A*. After encoding, we concatenate the embeddings of each GCN layer to get the output, denoted by $H_{f}$. As per GAE, we employ an inner product as our model’s decoder. The graph decoder generates a symmetric matrix *M* by implementing the inner-product on $H_{f}$ and then applying a sigmoid function. During training, *M* is required to approximate both the pre-defined graph *A* and the auxiliary graph $A_{s}$ (minimize $L_{ra}$ and $L_{rs}$, respectively). The bottom section is a module for clustering. $H_{f}$ serves as its input. The goal of introducing this module is to increase representations’ awareness of clustering. The module for clustering generates *Q* using a Student’s t-distribution. The clustering module creates a target distribution *P* by *Q* for the purpose of producing cluster-structured representations. By minimizing $L_{c}$ (KL divergence) between *Q* and *P*, the model can improve the cluster-friendliness of the representations.

The proposed model consists of the graph encoder, graph decoder, and clustering module, which will be introduced in turn as follows. Figure 1 is the flow chart of our proposed method.

### 3.1. Problem Definition

Given an undirected graph $G=\left(V,E\right),V=\left\{v_{1},v_{2}..v_{n}\right\}$ is a set of nodes, and |V|=n. *E* is the edge set. $X^{T}=\left[x_{1},x_{2},..x_{n}\right]\in R^{dxn}$ denotes a feature matrix of nodes. $A\in R^{nxn}$ denotes a symmetric adjacent matrix that indicates the connection of nodes, i.e., if node *i* links node *j*, then $A_{ij}=A_{ji}=1$, otherwise, $A_{ij}=A_{ji}=0$, i,j∈{1,2..n}, $A_{ij}\in \left\{0,1\right\}$. We define *D* as the degree matrix of *A*. $D_{ii}=A_{i1}+A_{i2}+\ldots +A_{in}$ and $D_{ij}=0$ when i≠j. More notations are summarized in Table 1.

### 3.2. Graph Encoder

GCN is used as a powerful tool for extracting features by integrating topological information into node attributes. In our model, we use the GCN as a basic module for encoding.

In GCN, nodes’ features are filtered in the frequency domain. As a result, the filtered features are supposed to be robust for being enhanced by their neighbors. After filtering, the features are transformed linearly by a weight matrix with an activation function. This process is formulated as the following equation:

(1) $$H_{1}=\phi \left({\tilde{D}}^{-1}\tilde{A}H_{0}W_{1}\right)\;$$

(2) $$H_{2}=\phi \left({\tilde{D}}^{-1}\tilde{A}H_{1}W_{2}\right)$$

$H_{0}$ denotes the input of the encoder, $H_{0}=X$. l∈{0,1,2,…,L} denotes the index of the layer, and *L* denotes the index of the last layer in the encoder. $W_{l}$ is the parameter of $l_{th}$—layer. ϕ denotes an activation function such as Tanh or LeakRelu. $\hat{A}=I+A$, ${\hat{D}}_{ii}=\sum_{j}{\hat{A}}_{ij}$. In GCN, different layers are supposed to generate features of different scales. It is supposed that the embeddings from fusing features from multiple layers should be more discriminative than those embeddings from the single layer. We apply a fusion strategy to the encoder, i.e., we simply concatenate each layer of the encoder for generating robust representations, and this operation can be formulated as the following equation:

(3) $$H_{f}=Concat\left(H_{1},H_{2}\right)\;$$

In (2), Concat denotes a concatenate function. $H_{1}\in R^{n\times d_{1}}$, $H_{2}\in R^{n\times d_{2}}$, $H_{f}\in R^{n\times d_{f}}$, $f=d_{1}+d_{2}$.

### 3.3. Graph Decoder

A graph decoder is usually used to reconstruct the original graph. Following Graph Auto-Encoder [15], we use an inner-dot operation as a graph decoder. The output of the decoder is a symmetric matrix that is constructed by the output of the encoder.

(4) $$M=\sigma ({H_{f}}^{T}\cdot H_{f})$$

σ is an activative function that scales the values to the range of (0,1). *M* is seen as the recovery of the original graph.

### 3.4. Optimization by Reconstructing Graphs

For the purpose of revealing the relationship between nodes thoroughly, we need to find the latent relationship between nodes that are missed by the original graph. To achieve this, we use *M* to reconstruct the original graph and the complementary graph simultaneously.

#### 3.4.1. Optimization by Reconstructing Original Graph

After obtaining *M* from the graph decoder, we optimize the model by minimizing the reconstruction loss between *M* and $\tilde{A}$:

(5) $$L_{ra}=\frac{1}{2n}{∥M-\tilde{A}∥}_{F}^{2}$$

This process is widely used in a graph auto-encoder model. Here we minimize the loss between *M* and the original graph to keep the performance in a basic level.

#### 3.4.2. Optimization by Reconstructing Complementary Graph

There are three parts to this optimization process. We describe them in the following sections: graph build, graph process, and minimization of reconstruction loss.

- **Graph Build** To make a complement to the given graph, we build a graph based on some similarity metric such as cosine similarity, which can discover the latent relationships between nodes in a global view. The complementary graph is constructed by the following equations:
  (6) $$S_{ij}=\frac{X_{i}X_{j}}{\left|\right|X_{i}{\left|\right|}_{2}\left|\right|X_{j}{\left|\right|}_{2}}$$
  (7) $${\tilde{S}}_{ij}=\frac{S_{ij}}{\sum_{k=1}^{n}S_{ik}}$$
  After calculating the similarity between each pair of nodes, we obtain a graph capturing the global relationships.
- **Graph Process** After graph building, we obtain an initial graph S that unavoidably contains noise. To obtain a relatively clean graph, we need to filter noise. We introduce a simple but effective filtering mechanism.
  (8) $$S_{i}^{rank}=sort\left({\tilde{S}}_{i}\right)\;$$
  (9) $$A_{s_{ij}}=\left\{\begin{array}{ccc} {\tilde{S}}_{ij} & \; & if\;{\tilde{S}}_{ij}\geq {\tilde{S}}_{ir_{K}}\\ \; & \\ 0 & \; & else \end{array}\right.$$
  At first, we rank each row of *S* in descending order by a sort function. After ranking, $S_{i}^{rank}=\left\{{\tilde{S}}_{ir_{1}},{\tilde{S}}_{ir_{2}},{\tilde{S}}_{ir_{3}},\ldots ,{\tilde{S}}_{ir_{n}}\right\},{\tilde{S}}_{ir_{k}}\geq {\tilde{S}}_{ir_{k+1}}$. And then, by using a filter mechanism, we only keep relations of top-K highest confidence, and we reduce the rest to 0 to decrease the impact of false relations.
- **Minimization of reconstruction loss** After the process of filtering, we obtain a more reliable graph $A_{s}$. And we implement representation learning by minimizing the loss between *M* and $A_{s}$, which is formulated as:
  (10) $$L_{rs}=\frac{1}{2n}{∥M-A_{s}∥}_{F}^{2}$$
  The $\tilde{A}$ and $A_{s}$ are supervisors that are complementary to each other.

#### 3.4.3. The Joint Reconstruction Loss

A single supervisor may lead to bias in representation learning. Instead of using one single supervisor, we minimize the reconstruction loss by both supervisors $\tilde{A}$ and $A_{s}$. The objective function is formulated as follows:

(11) $$L_{rec}=\lambda L_{rs}+L_{ra}\;$$

λ is a hyper-parameter used to control the importance of $L_{rs}$.

### 3.5. Clustering Module

For unsupervised learning approaches, there are no given labels for target functions. We need an optimization that can be used to guide our model to facilitate clustering tasks. As most graph clustering models do, we introduce an alternative strategy to conduct a clustering-oriented optimization. We use Student’s t-distribution as the kernel to measure the similarity between centroids and embeddings:

(12) $$q_{ij}=\frac{(1+∥h_{i}-\mu_{k}{{∥}^{2})}^{-1}}{\sum_{t}(1+∥h_{i}-\mu_{t}{{∥}^{2})}^{-1}}$$

$\mu_{k}$ denotes the centroid of cluster k. It is initialized by k-means or random vectors. $q_{ij}$ denotes the probability that node *i* belongs to cluster *j*. To improve the accuracy of centroids, we generate a target distribution. By matching the Student’s t-distribution of *Q* to the target distribution of *P*, the clusters’ centroids and embeddings are simultaneously optimized. The target distribution is constructed by the following equation:

(13) $$p_{ij}=\frac{q_{ij}^{2}/f_{j}}{\sum_{k}q_{ik}^{2}/f_{k}}$$

In (11), $f_{k}=\sum_{i}q_{ik}$. And the optimizing process is to minimize the KL divergence loss between $q_{ij}$ and $p_{ij}$:

(14) $$L_{c}=KL\left(P∥Q\right)=\sum_{i}\sum_{j}p_{ij}log\frac{p_{ij}}{q_{ij}}$$

### 3.6. Joint Optimization

To train the graph encoder-decoder and clustering module jointly, we design the objective function as:

(15) $$L=L_{c}+L_{rec}$$

$L_{c}$ denotes the clustering loss, and $L_{rec}$ denotes the reconstruction loss. After training, we can obtain the clustering results *Y* from *Q*, and the prediction of node *i* is assigned by:

(16) $$y_{i}=\underset{c}{\arg\max}q_{ic}\;$$

Specifically, $Y=[y_{1},y_{2},...,y_{n}]$, $y_{i}$ is the position of the max value in $q_{i}$, which is a pseudo label of cluster as well. The detailed steps are summarized in Algorithm 1.
**Algorithm 1** Deep Graph Clustering via Graph Augmentation**Require**:
  Attribute matrix *X*, adjacent matrix *A*, iteration number iter, hyperparameter λ,K
**Ensure:**
  Clustering result *Y*;
Construct Top-K similarity matrix $A_{s}$**for**t=1 to iter **do**   Generate the embeddings $h_{1},h_{2}$ by (1),(2)   Generate $h_{f}$ by (3)   Construct *M* by (4)   Calculate reconstruction loss by (11)   Generate *Q* by (12)   Generate *P* by (13)   Calculate clustering loss by (14)   Update the whole framework by (15)**end for**Obtain *Y* by (16).**return***Y*;


### 3.7. Complexity Analysis

For the sparsity of the matrix, the computational complexity of GCN is linear with |E|. Let *d* be the maximum number of neurons in hidden layers, the complexity is $O(|E|d^{2})$. In addition, we let k be the number of clusters, and the computational complexity of (10) is O(nk+nlogn). Taking both GCN and clustering module into account, the final complexity is $O(|E|d^{2}+nk+nlogn)$.

## 4. Experiment

### 4.1. Datasets

We implement experiments on four widely used graph datasets. More details about them are summarized in Table 2.

- **Citeseer** This is a citation dataset. Papers in it are divided into six categories: Agents, Artificial Intelligence, Database, Information Retrieve, Machine Language, HCI. Each edge represents a citation relationship between documents. Each node denotes a paper whose feature is represented by a {0, 1} vector. Each dimension is a keyword from a specific vocabulary.
- **Dblp** It is a cooperative network. Authors in it are divided into four classes: database, data mining, machine learning, and information retrieval. An edge represents a cooperative relationship between authors. The node features are the elements of a bag-of-words represented by keywords.
- **Acm** It is a paper network. An edge between nodes represents that these two papers are written by the same author. Papers are divided into three classes: Database, Wireless Communication, and Data Mining. The features are bag-of-words of keywords from corresponding areas.
- **Pubmed** It is a citation dataset about Diabetes. The publications in it are divided into 3 classes: Diabetes Experimental, Diabetes type1, and Diabetes type2. Each node is represented by a tf-idf vector of keywords.

### 4.2. Baselines

We compare our proposed method with 12 methods which can be divided into 4 types: Non-model based, Auto-Encoder based, Graph Auto-Encoder based, and Hybrid-module based.

- **K-means** A widely used clustering algorithm based on an EM [36] updating strategy.
- **AE** [10] A classical Deep model for unsupervised learning.
- **DEC** [11] A deep embedding model based on Auto-Encoder for clustering.
- **IDEC** [9] A deep model based on DEC with an additional Auto-Encoder module for preserving the local structure of data.
- **GAE&VGAE** [15] A GCN-based model for unsupervised learning, based on the frameworks of AE&VAE.
- **ARGE&ARVGE** [16] A GAE (VGAE) based model, with the adversarial training strategy to regularize the distribution of embedding for robust representations.
- **DAEGC** [19] An attention mechanism based graph clustering model. Instead of being guided by a given graph structure, it learns to aggregate by posing attention scores on each neighbor.
- **SDCN** [20] A hybrid deep clustering model that integrates embeddings from both Auto-Encoder and GCN module, which is designed for easing the problem of over-smoothness.
- **AGCN** [34] Based on SDCN, it proposed a method to learn an attention mechanism to fuse the embeddings from different modules reasonably.
- **DFCN** [21] Based on SDCN, it introduces a cross-modality fusion mechanism to improve the robustness.

### 4.3. Parameter Settings

As most GCN based models do, we use a 2-layer network for our model. Dimensions of each layer are *d*-256-16. Specifically, *d* is the dimension of input. The training process is divided into two steps. In the first step, we pre-train the network without the clustering module to minimize the reconstruction loss of similarity and graph structure. In the second step, together with cluster loss, we train the whole network. After analyzing the effect of hyperparameters, we set λ=0.1 for Citeseer and λ=10 for the other. Also, we set K=100 for top-K similarity. For Citeseer, Dblp, and Acm, we set the learning rate to 0.001, for Pubmed, we set it to 0.005. For Dblp and Pubmed, we train the network for 500 epochs, and 100 epochs for Acm, 400 epochs for Citeseer. For fairness, we set the dimension of the network of GAE&VGAE the same as ours. In addition, for dealing with Pubmed, we use a sampling strategy for training. The sampling rate is set to 0.25 in our experiment. In each epoch, we sample a subgraph that contains 25% nodes of the dataset for training. For AE, GAE&VGAE, we use K-means to obtain the clustering results. For clustering methods, we follow the settings of their corresponding papers. We repeat the experiment 10 times to obtain the average result, which shows in Table 3. All experiments are implemented with PyTorch and run on a GPU (GeForce GTX 1080Ti).

### 4.4. Metrics

We use four popular metrics to evaluate the clustering performance: ACC (Accuracy), NMI (Normalized Mutual Information), ARI (Average Rank index), and F1 (macro F1-score). ACC is obtained by counting the matching pairs of predictions and labels and calculating the ratio of correctly matched pairs in the total matchings. NMI is used to measure the mutual information between prediction and true labels. ARI is used to measure the decision of clustering. F1 is an overall measurement for precision and recall. Higher values denote better performance.

### 4.5. Analysis of Result

In our experiments, our method was compared with 12 other methods on four benchmark datasets. Table 3, Table 4, Table 5 and Table 6 show the results. **Bold** numbers represent the best performance, the underline denotes the second best. From these tables, we have these observations:

- We can observe from these tables that the proposed method outperforms all the compared baseline methods on four benchmark datasets on most metrics. For example, in Dblp, our model outperforms the second-best one by nearly 4 pp (pp: percentage point), 7 pp, 8 pp, 5 pp on ACC, NMI, ARI, and F1 respectively. In Pubmed, compared to the second strongest, our model outperforms it by nearly 2 pp, 3 pp, 3 pp, 2 pp on ACC, NMI, ARI, F1 respectively. There are three reasons for the effectiveness of our model: First, we fuse embeddings from multiple layers to generate discriminative representations; Second, we construct a filtered graph from the original feature space to preserve the global relations of nodes; Last, we develop a joint training strategy to learn representations that can facilitate clustering and preserve both local relations and intrinsic global relations of nodes.
- AE, DEC, and IDEC only use node features for generating embeddings, which leads to a sub-optimal clustering performance compared with GCN-based models. K-means clustering is directly performed in the original feature space, it can be used to measure the quality of features. From k-means, we can observe that the quality of data in Acm is the best.
- In GAE, VGAE, ARGE, and ARVGE, they generate embeddings from a single layer. Compared with them, besides reconstructing intrinsic relationships, our model can fuse multi-scale features to strengthen the discriminativeness for embeddings.
- DAEGC exploited the attention mechanism for aggregating. Although considering the relations between nodes in a wider range, it implements representation learning by the supervision from the given graph structure, which cannot exploit the hidden relations that are missed by the given graph. Compared with it, our model has two advantages: First, we explore relations from a global view. Second, the explored relations come from original space, which can be considered to be more intrinsic.
- SDCN, AGCN, and DFCN are powerful deep clustering models that exploit multi-modality to generate discriminative embeddings. Regardless of alleviating the problem of over-smoothness, these models fail to explore the latent relations of nodes that cannot be observed from the given graph. However, by measuring the similarity between nodes, our model successfully revealed the missing relations from the original feature space and outperforms the mentioned models.

### 4.6. Ablation Study

To make it clear how each part contributes to the proposed model, we implement experiments by removing them. Also, we conduct experiments to validate the strategy of fusing embeddings of each layer to improve the representations. The results of these experiments are shown in Table 7 and Table 8, respectively.

#### 4.6.1. The Effectiveness of Each Component

Table 7 illustrates how each component of the model influences its performance. No single component of the model can outperform the other two across all datasets. In Citeseer and Pubmed, deleting the fusion portion has the most significant effect on performance. We conclude that the feature from various scales strengthens the representations in these datasets. However, in Dblp, similarity supervision has the greatest impact, indicating that the effectiveness of mining latent edges is promising. The combination of similarity and fusion dominates the performance of Citeseer, whereas the combination of similarity and adjacent dominates the performance of Dblp. Compared to other datasets, however, it appears that only the incorporation of three-part data can result in significant improvements for Acm and Pubmed.

#### 4.6.2. The Effectiveness of Each Layer

To demonstrate the efficacy of the fusion technique, we implement the clustering task on each layer individually. Table 8 provides the results. $H_{1}$ and $H_{2}$ represent embeddings from layer-1 and layer-2, respectively, whereas $H_{f}$ represents the combination of $H_{1},H_{2}$. We can observe that, across all datasets, the power of single-layer representation is consistently weaker than that of multiple-layer representation. In addition, we discovered that for varied datasets, individuals have varying preferences for the neighborhood scale. For Citeseer and Pubmed, embeddings of layer-1 are preferred, whereas embeddings of layer-2 improve clustering performance for Dblp and Acm. However, optimal performance can be achieved by combining embeddings from both layers, validating the efficacy of our fusion technique.

### 4.7. Analysis of Hyperparameters

In our experiments, we introduce 2 hyperparameters. K is the number of top-K nearest neighbors for target nodes. But it is used for choosing the top-K values of each row in the similarity matrix. λ is a hyper-parameter that is used for adjusting the importance of reconstruction of original relations of nodes.

#### 4.7.1. Analysis of λ

**Figure 2:**
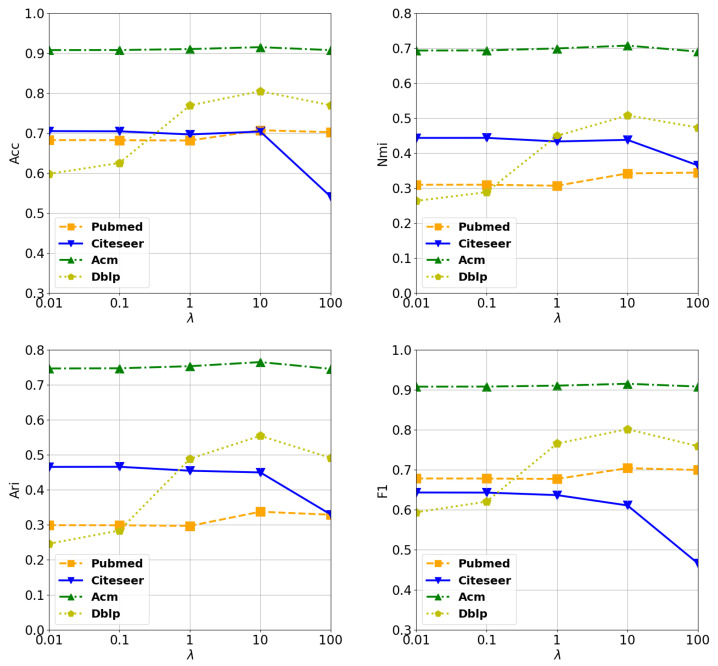
The sensitivity analysis of λ in (11).

We empirically choose the range of λ as {100,10,1,0.1,0.01}. In Acm, the fluctuation of the performance is slow and tiny, but it is clear to see that the best performance is achieved when λ=10. The best values for λ in Pubmed and Dblp is 10 too, as we can observe easily in Figure 2. However, the best performance is achieved in Citeseer when λ=0.1. These observations can validate that: (1) The auxiliary graph is helpful for clustering tasks. (2) Compared to Citeseer, the auxiliary graph plays more import roles in Pubmed, Dblp, and Acm. From the degree of improvement, Dblp is benefited most. It achieves an improvement of nearly 20% in Acc from 0.01 to 10. Although achieving improvement, the degree is not as much as Dblp’s. The reason may be that compared to the given graph of Dblp, graphs of the other datasets can cover relationships more completely. Also we can observe that the performance tend to decrease to different degrees for all datasets when λ varies from 10 to 100. There are two reasons for this: (1) Although filtered, the auxiliary graph still contains noise, and putting too much weight on it will increase the impact of noise. (2) There exists linked pairs in the given graph, they belong to the same cluster, but they are not linked in the auxiliary graph. Putting too much emphasis on the auxiliary graph may ignore this kind of relationship, which leads to a sub-optimal performance. According to the reasons above, we cannot put too much weight on the auxiliary graph during the training.

#### 4.7.2. Analysis of K

**Figure 3:**
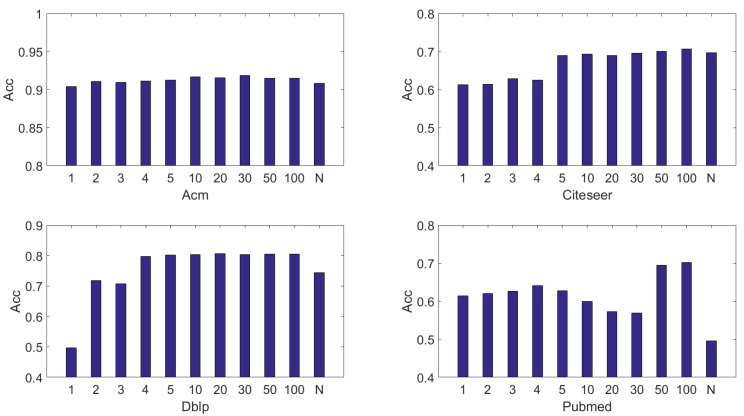
The sensitivity of hyperparameter K (the parameter of KNN in (9)).

The range of *K* is {1,2,3,4,5,6,7,8,9,10,20,30,50,100,N}. N denotes the number of nodes in dataset. First of all, from the Figure 3 it is not hard to observe that for all datasets the best performances are achieved when K=100. However, when K=N then all the performances decrease to different degrees. There exists too much noise in an unfiltered auxiliary graph that will harm the performance noticeably. First of all, for all datasets, the best choice for *K* is 100 according to the figure. For Acm, the performance always keeps stable when *K* varies. Although a little, the auxiliary graph still improves the performance. For Citeseer, Dblp, and Pubmed, the performance can be improved substantially when *K* reaches or passes a certain thresh. In our experiment, the thresh for Citeseer is 5, 4 for Dblp, 50 for Pubmed. In most cases, the performance increases as the *K* increases. However, when K=N, the performance become worse than K=100. This is because a full connected graph which is built by raw features contains much more noise than a filtered graph.

### 4.8. Study on the Influence of Graph Structure and Attribute

**Figure 4:**
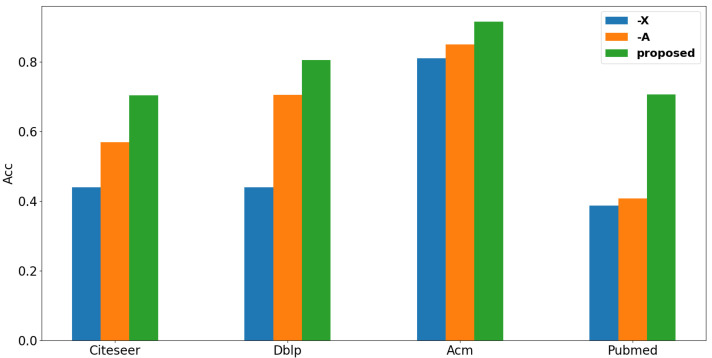
Impact to performance by structure and features.

To study how the structure influences our method, we conduct experiments in two different ways: (1) Remove the attribute from the input; (2) remove structure information from the input. The results are shown in Figure 4. From this figure we can easily observe that with the structure only our method can achieve better performance than the performance with features only. We can infer that for these datasets, the structure plays a more critical role than the feature does in our method. And we can easily observe that when we integrate attribute with structure as input, we can achieve the best performance over other methods that are compared in our experiments.

## 5. Conclusions

In this paper, we propose a clustering model termed Auxiliary Graph for Attribute Graph Clustering. In our model, we build an auxiliary graph to reveal the latent relations of nodes in a global view. To reduce the impact of inherent noises in datasets, we disregard unreliable relations by a filter mechanism. With the help of the auxiliary graph, our model can learn a more reliable representation. With the help of the fusion strategy and clustering module, the discriminativeness and clustering-awareness of learned representations are both improved. Experiments on four benchmark datasets demonstrate that our model can outperform state-of-the-art baselines in most cases. Although achieving promising performance, our model still has room to improve. In the future, we will improve our model to fit different datasets, especially large-scale datasets. 

## Figures and Tables

**Table 1:** Notations.

Notations	Meaning
$X\in R^{d\times n}$	Feature matrix
$A\in R^{n\times n}$	Adjacent matrix
$I\in R^{n\times n}$	Identity matrix
$A_{s}\in R^{n\times n}$	Filtered similarity matrix
$\hat{A}\in R^{n\times n}$	Adjacent matrix with self-loop
$\tilde{A}\in R^{n\times n}$	Normalized adjacent matrix
$A_{s}\in R^{n\times n}$	Constructed similarity matrix
$D\in R^{n\times n}$	Degree matrix
$H_{f}\in R^{d\times n}$	Output of graph encoder
$M\in R^{n\times n}$	Reconstructed matrix
$S\in R^{n\times n}$	Constructed similarity matrix
$Q\in R^{n\times K}$	Soft assignment distribution
$P\in R^{n\times K}$	Target distribution

**Table 2:** Benchmark Datasets.

Dataset	Nodes	Dimension	Clusters	Edges	Degree
Citeseer	3327	3703	6	4732	99
Dblp	4058	334	4	7056	45
Acm	3025	1870	3	26,256	90
Pubmed	19,717	500	3	44,325	142

**Table 3:** Clustering results on Citeseer.

Method	ACC	NMI	ARI	F1
k-means	55.06	29.21	24.56	53.03
AE	53.93	27.56	26.03	50.53
DEC	60.96	33.36	33.20	57.13
IDEC	63.16	36.54	36.75	60.37
GAE	60.55	36.34	35.50	56.24
VGAE	51.41	28.96	24.88	49.48
ARGE	54.40	26.10	24.50	52.90
ARVGE	57.30	35.00	34.10	54.60
DAEGC	64.54	36.41	37.78	62.20
SDCN	65.96	38.71	40.157	63.62
AGCN	68.79	41.54	43.79	62.37
DFCN	69.50	43.90	45.50	**64.30**
AGAGC	**70.46**	**44.36**	**46.56**	64.28

**Table 4:** Clustering results on Pubmed.

Method	ACC	NMI	ARI	F1
k-means	59.83	31.05	28.1	58.88
AE	63.07	26.32	23.86	64.01
DEC	60.154	22.44	19.55	61.49
IDEC	60.70	23.67	20.58	62.41
GAE	62.09	23.84	20.62	61.37
VGAE	68.48	30.61	30.155	67.68
ARGE	65.26	24.8	24.35	65.69
ARVGE	64.25	23.88	22.82	64.51
DAEGC	68.73	28.26	29.84	68.23
SDCN	64.20	22.87	22.30	65.01
AGCN	63.61	23.31	22.36	64.19
DFCN	68.89	31.43	30.64	68.10
AGAGC	**70.77**	**34.33**	**33.81**	**70.46**

**Table 5:** Clustering results on Dblp.

Method	ACC	NMI	ARI	F1
k-means	38.35	10.99	6.68	32.10
AE	38.62	14.03	7.41	31.72
DEC	61.46	27.53	25.25	61.82
IDEC	55.92	24.56	18.37	56.82
GAE	53.42	29.29	16.83	54.9
VGAE	53.06	28.87	16.65	54.34
ARGE	64.44	30.21	26.21	64.32
ARVGE	61.94	25.63	23.91	60.57
DAEGC	62.05	32.49	21.03	61.75
SDCN	68.05	39.50	39.15	67.71
AGCN	73.26	39.68	42.49	72.80
DFCN	76.00	43.70	47.00	75.70
AGAGC	**80.50**	**50.77**	**55.41**	**80.16**

**Table 6:** Clustering results on Acm.

Method	ACC	NMI	ARI	F1
k-means	68.17	33.40	31.29	68.42
AE	78.55	44.53	46.98	78.69
DEC	72.52	43.50	43.48	70.60
IDEC	78.33	50.83	51.52	76.44
GAE	89.06	64.69	70.47	89.05
VGAE	76.78	43.33	41.14	76.96
ARGE	83.06	49.31	55.77	84.81
ARVGE	83.65	52.11	57.08	81.40
DAEGC	86.94	56.18	59.35	87.07
SDCN	90.45	68.31	73.91	90.42
AGCN	90.59	68.38	74.20	90.58
DFCN	90.90	69.40	74.90	90.80
AGAGC	**91.50**	**70.74**	**76.49**	**91.51**

**Table 7:** Compare the impact of removing each part of the model.

Dataset	Remove	ACC	NMI	ARI	F1
Citeseer	w/o S	65.29	40.26	40.15	58.81
	w/o C	57.24	36.82	31.76	50.26
	w/o A	70.35	43.70	45.12	62.09
	Proposed	**70.46**	**44.36**	**46.56**	**64.28**
Acm	w/o S	90.54	68.50	74.04	90.58
	w/o C	89.87	67.12	72.51	59.87
	w/o A	88.96	65.65	69.95	89.07
	Proposed	**91.60**	**70.74**	**76.49**	**91.51**
Dblp	w/o S	59.03	25.55	23.64	58.58
	w/o C	80.11	50.31	55.11	79.68
	w/o A	77.64	49.05	49.37	77.70
	Proposed	**80.50**	**50.77**	**55.41**	**80.16**
Pubmed	w/o S	62.15	24.78	21.40	62.14
	w/o C	39.95	-	-	19.04
	w/o A	55.60	18.20	14.53	54.31
	Proposed	**70.77**	**34.33**	**33.81**	**70.46**

**Table 8:** The clustering performance on each layer.

Dataset	Layer	ACC	NMI	ARI	F1
Citeseer	$H_{1}$	67.76	41.80	42.29	63.73
	$H_{2}$	67.95	41.56	40.93	59.52
	$H_{f}$	**70.46**	**44.36**	**46.56**	**64.28**
Dblp	$H_{1}$	78.11	47.81	51.30	77.36
	$H_{2}$	80.08	50.15	55.11	79.62
	$H_{f}$	**80.50**	**50.77**	**55.41**	**80.16**
Acm	$H_{1}$	89.60	66.98	71.65	89.67
	$H_{2}$	90.62	69.19	74.41	90.63
	$H_{f}$	**91.50**	**70.74**	**76.49**	**91.51**
Pubmed	$H_{1}$	63.73	26.03	24.37	64.98
	$H_{2}$	62.91	21.34	20.63	63.31
	$H_{f}$	**70.77**	**34.33**	**33.81**	**70.46**

## Data Availability

Data available on request from the authors.
